# Defining Body Fatness in Adolescents: A Proposal of the Afad-A Classification

**DOI:** 10.1371/journal.pone.0055849

**Published:** 2013-02-06

**Authors:** María del Mar Bibiloni, Antoni Pons, Josep A. Tur

**Affiliations:** 1 Research Group on Community Nutrition and Oxidative Stress, University of Balearic Islands, Palma de Mallorca, Spain; 2 CIBERobn (Fisiopatología de la Obesidad y la Nutrición CB12/03/30038), Instituto de Salud Carlos III, Spain; Tulane School of Public Health and Tropical Medicine, United States of America

## Abstract

**Aims:**

Body mass index (BMI) shows several limitations as indicator of fatness. Using the International Obesity Task Force (IOTF) reference and the World Health Organization (WHO) standard 2007 on the same dataset yielded widely different rates. At higher levels, BMI and the BMI cut-offs may be help in informing a clinical judgement, but at levels near the norm additional criteria may be needed. This study compares the prevalence of overweight and obesity using IOTF and WHO-2007 references and interprets body composition by comparing measures of BMI and body fatness (fat mass index, FMI; and waist-to-height ratio, WHtR) among an adolescent population.

**Methods and Results:**

A random sample (n = 1231) of adolescent population (12–17 years old) was interviewed. Weight, height, waist circumference, triceps and subscapular skinfolds were used to calculate BMI, FMI, and WHtR. The prevalence of overweight and obesity were 12.3% and 15.4% (WHO standards) and 18.6% and 6.1% (IOTF definition). Despite that IOTF cut-offs misclassified less often than WHO standards, BMI categories were combined with FMI and WHtR resulting in the Adiposity & Fat Distribution for adolescents (AFAD-A) classification, which identified the following groups normal-weight normal-fat (73.2%), normal-weight overfat (2.1%), overweight normal-fat (6.7%), overweight overfat (11.9%) and obesity (6.1%), and also classified overweight at risk and obese adolescents into type-I (9.5% and 1.3%, respectively) and type-II (2.3% and 4.9%, respectively) depending if they had or not abdominal fatness.

**Conclusions:**

There are differences between IOTF and WHO-2007 international references and there is a misclassification when adiposity is considered. The BMI limitations, especially for overweight identification, could be reduced by adding an estimate of both adiposity (FMI) and fat distribution (WHtR). The AFAD-A classification could be useful in clinical and population health to identify overfat adolescent and those who have greater risk of developing weight-related cardiovascular diseases according to the BMI category.

## Introduction

In children and adolescents, the body mass index (BMI) for age has been established as the main measurement to define overweight and obesity, because it can be easily obtained and is correlated with percentage of body fat [1].

Despite some discussion, in epidemiological studies is general agreement on the appropriateness of BMI to define overweight and obesity with an international standard [2]. Two international references are widely used: the International Obesity Task Force (IOTF) reference and the World Health Organization (WHO) standard 2007. The IOTF reference for children and adolescents 2–18 years old [3] was developed from a database of 97,876 boys and 94,851 girls from birth to 25 years from six countries (Brazil, Great Britain, Hong Kong, the Netherlands, Singapore and the USA). Centile curves were constructed using the LMS method, and BMI values of 25 and 30 at 18 years of age for boys and girls were tracked back to define BMI values for overweight and obesity at younger ages. The WHO-2007 standard for children and adolescents (5–19 years old) [4] was developed using the 1977 National Center for Health Statistics (NCHS)/WHO growth reference by addressing its limitations and linking construction to the WHO Child Growth Standards curves for children under five years old. Data points for children and adolescents with measurements suggestive of high adiposity were excluded. The total simple size used to generate the curves was 22,917 children. State of the art statistical techniques were used to construct and smooth the new growth curves.

Nevertheless, it became clear that using IOTF or WHO references on the same dataset yielded widely different rates [2], [5]–[7]. Moreover, there are some limitations associated with the use of BMI as indicator of fatness, as follows: individuals with increased muscle mass may also have increased BMI; and also individuals with decreased lean body mass and increased adiposity may be misclassified by assessment with BMI; BMI fails to assess the accumulation of abdominal fat, which mainly increases the risk of diabetes, hypertension and cardiovascular diseases (CVD) risks; and BMI is relatively insensible to body composition changes [8].

At higher levels, BMI and the BMI cut-offs may be help in informing a clinical judgement, but at levels near the norm additional criteria may be needed [9], such as skinfold thickness [10] or waist circumference (WC). At the upper end of the BMI distribution curve [11], an important percentage of individuals classified as overweight or obese may not have excess fat [12]. Lately, it has been described [13] in adults a new syndrome named normal-weight obese (NWO), which has been associated with an unfavourable lipid and inflammatory profile. Accurate estimation of body fatness is essential not only for the prevention and treatment of CVD risk factors, but also in psychosocial complications related to body dissatisfaction which are especially prevalent among adolescents [14]. We thus decided to carry out additional analysis to better explore this issue. The aims of this study were to compare the prevalence of overweight and obesity using the IOTF and the WHO-2007 references and interpret body composition by comparing measures of body weight (BMI) and body fatness (fat mass index, FMI; and waist-to-height ratio, WHtR) among an adolescent population.

## Methods

### Study Design

The study is a population-based cross-sectional nutritional survey carried out in the Balearic Islands (2007–2008).

### Selection of Participants, Recruitment and Approval

A multicenter study was performed on Balearic Islands’ adolescents aged 12–17 years. The population was selected by means of a multiple-step, simple random sampling, taking into account first the location (Palma de Mallorca, Calvià, Inca, Manacor, Maó, Eivissa, Llucmajor, Santa Margalida, S’Arenal, Sant Jordi de Ses Salines) and then by random assignment of the schools within each city. Sample size was stratified by age and sex. The socioeconomic variable was considered to be associated to geographical location and type of school. As the selection of schools was done by random selection and fulfilling quota, this variable was also considered to be randomly assigned.

To calculate the number of adolescents to be included in the study in order to guarantee a representative sample of the whole Balearic Islands, we selected the variable with the greatest variance for this age group from the data published in the literature at the time the study was planned; that was BMI [15]. The sampling was determined for the distribution of this variable; the confidence interval (CI) was established at 95% with an error ±0.25. The established number of subjects was 1,500. The total number of subjects was uniformly distributed in the cities and proportionally distributed by sex and age group.

The sample was oversized to prevent loss of information and as necessary to do the fieldwork in complete classrooms. In each school, all the adolescents of one classroom were proposed to participate in the survey. A letter about the nature and purpose of the study informed parents or legal tutors. After receiving their written consent, the adolescents were considered for inclusion in the study. After finishing the field study, the adolescents who did not fulfil the inclusion criteria were excluded. Finally, the sample was adjusted by a weight factor in order to balance the sample in accordance to the distribution of the Balearic Islands’ population and to guarantee the representativeness of each of the groups, already defined by the previously mentioned factors (age and sex). The final number of subjects included in the study was 1,231 adolescents (82% participation). The reasons to not participate were (a) the subject declined to be interviewed, and (b) the parents did not authorize the interview.

This study was conducted according to the guidelines laid down in the Declaration of Helsinki, and all procedures involving human subjects were approved by the Balearic Islands’ Ethics Committee (Palma de Mallorca, Spain) no. IB-530/05-PI. Written informed consent was obtained from all subjects and also from the next of kin, caretakers, or guardians on the behalf of the minors participants involved in the study.

### Anthropometric Measurements

Height was determined using a mobile anthropometer (Kawe 44444, Asperg, Germany) to the nearest millimetre, with the subject’s head in the Frankfurt plane. Body weight was determined to the nearest 100 g using a digital scale (Tefal, sc9210, Rumilly, France). The subjects were weighed in bare feet and light underwear. The following circumferences were measured using a non-stretchable measuring tape (Kawe, 43972, France): mid-upper arm circumference (MUAC), WC, hip circumference (HC) and thigh circumference (TC). The subjects were asked to stand erect in a relaxed position with both feet together on a flat surface. MUAC was measured with as the midpoint of the length of the humorous. WC was measured as the smallest horizontal girth between the costal margins and the iliac crests at minimal respiration. HC was taken as the greatest circumference at the level of greater trochanters (the widest portion of the hip) on both sides. TC was measured below the gluteal fold. Measurements were made to the nearest 0.1 cm. Triceps and subscapular skinfold thickness (ST) were measured at the right side of the using a Holtain skinfold calliper (Tanner/Whitehouse, Crosswell, Crymych, UK), and the mean of three measurements was used. Body fat percentage (%BF) was measured from triceps and subscapular ST according to Slaughter et al. [16]. This equation has been proposed as the most accurate for estimation of %BF from ST in this particular population of adolescents [17]. Height and weight measures were used to calculate BMI (kg/m^2^) and WC and height were used to calculate waist-to-height ratio (WHtR). %BF and height were used to calculate fat mass index (FMI; kg/m^2^).

#### Overweight and obesity definition

Overweight and obesity were determined based on age- and sex-specific BMI cut-offs as followed: 1) 85th percentile for overweight and 95th percentile for obesity using the WHO growth standards for children and adolescents [4], and 2) the cut-offs developed and proposed for international comparisons by Cole et al [3], recommended for use also by IOTF.

#### Normal-fat and overfat definition

Normal-fat and overfat were determined using sex-specific cut-offs [18] for adolescents: 4.58 kg/m^2^ in boys and 7.76 kg/m^2^ in girls.

#### Abdominal obesity definition

A WHtR cut-off of 0.5 was used to define abdominal obesity for both boys and girls [19].

### Statistics

Analyses were performed with Statistical Package for the Social Sciences version 19.0 (SPSS, Inc., Chicago, IL, USA). Significant differences in prevalence were calculated by means of χ^2^. Differences between groups’ means were tested using ANCOVA adjusted by age. The level of significance was established for *P* values <0.05.

## Results

Adolescents were classified according to their body weight by BMI using WHO-2007 and IOTF cut-offs, overall adiposity by FMI, and presence or absence of abdominal obesity by WHtR (Table 1). Large differences in overweight and obesity prevalence were obtained when results using WHO-2007 and IOTF cut-offs were compared. While obesity prevalence was higher using WHO-2007 (15.4%) than IOTF (6.1%) references; overweight prevalence was higher using IOTF (18.6%) than WHO-2007 (12.3%) cut-offs. Overall excessive weight (overweight and obesity) was higher by WHO-2007 (27.7%) than IOTF (24.7%). Nevertheless, results showed that using both BMI cut-offs the prevalence of excessive weight was higher than the percentage of overfat adolescents (19.8%) and adolescents with abdominal obesity (7.7%).

**Table 1:** Prevalence (%) of overweight and obesity (by BMI), adiposity (by FMI) and abdominal obesity (by WHtR) among the Balearic Islands’ adolescents, Spain (2007–2008).

Anthropometric variable			Cut-offs	Total(%)	Boys(%)	Girls(%)	*P*
BMI (kg/m^2^)	IOTF	Normal-weight	BMI-for age and sex<25 kg/m^2^	75.3	72.3	77.8	<0.050
		Overweight	BMI-for age and sex ≥25–<30 kg/m^2^	18.6	21.2	16.4	<0.050
		Obesity	BMI-for age and sex ≥30 kg/m^2^	6.1	6.5	5.8	NS
	WHO-2007	Normal-weight	BMI-for age and sex <P85	72.3	67.7	76.1	<0.010
		Overweight	BMI-for age and sex ≥P85–<P95	12.3	13.9	10.9	NS
		Obesity	BMI-for age and sex ≥P95	15.4	18.3	13.0	<0.050
FMI (kg/m^2^)		Normal-fat	Boys: FMI<4.58 kg/m^2^	80.2	72.3	86.8	<0.001
			Girls: FMI<7.76 kg/m^2^				
		Overfat	Boys: FMI≥4.58 kg/m^2^	19.8	27.7	13.2	<0.001
			Girls: FMI≥7.76 kg/m^2^				
WHtR		Absence of abdominal obesity	WHtR<0.5	92.3	91.6	92.9	NS
		Abdominal obesity	WHtR≥0.5	7.7	8.4	7.1	NS

Abbreviations: BMI, body mass index; FMI, fat mass index; WHtR, waist-to-height ratio; IOTF, International Obesity Taskforce; WHO, World Health Organization. Significant differences (boys vs. girls) by χ^2^. NS: not significant.

Table 2 shows the prevalence of normal-weight, overweight and obesity according to overall adiposity (FMI) and abdominal fatness (WHtR) cut-offs. Thus, the three body weight groups obtained by IOTF and WHO-2007 cut-offs (normal-weight, overweight and obesity) were subgrouped as follows: first, according to presence or absence of overfat; and then, according to presence or absence of abdominal obesity. Results showed that almost all obese adolescents (95.1%) were overfat by IOTF cut-offs, while 14.8% of adolescents classified by the WHO-2007 cut-offs as obese were normal-fat (2.3% of population). The overweight overfat prevalence was 11.9% by IOTF and 5.1% by WHO-2007 cut-offs, whereas overweight normal-fat prevalence was 6.7% and 7.1%, respectively. The prevalence of overweight normal-fat was higher among girls than boys using the IOTF cut-offs (*P*<0.001). Results also showed that the prevalence of normal-weight overfat adolescents was 2.1% by IOTF cut-offs and 1.5% by WHO cut-offs, which was higher in boys than girls independently of the cut-off used. Then, 20.2% of overweight overfat adolescents by IOTF cut-offs and 5.9% by WHO-2007 cut-offs had abdominal obesity, while these percentages increased to 78.7% and 46.1% in obese adolescents, respectively.

**Table 2:** Prevalence (%) of normal-weight, overweight and obesity using different indicators for the condition in Balearic Islands’ adolescent population, Spain (2007–2008).

Body composition markers		Total		Boys		Girls		*P* ^3^	*P* ^4^
Categories	Cut-offs	WHO-2007^1^	IOTF^2^	WHO-2007^1^	IOTF^2^	WHO-2007^1^	IOTF^2^		
**Normal-weight^1,2^**									
Normal-fat	FMI<4.58/7.76 kg/m^2^ (boys/girls)	70.8	73.2	64.9	68.3	75.8	77.4	<0.001	<0.010
Overfat	FMI≥4.58/7.76 kg/m^2^ (boys/girls)	1.5	2.1	2.9	4.0	0.3	0.5	<0.001	<0.001
**Overweight^1,2^**									
Normal-fat	FMI<4.58/7.76 kg/m^2^ (boys/girls)	7.1	6.7	6.1	3.6	8.0	9.3	NS	<0.001
Overfat	FMI≥4.58/7.76 kg/m^2^ (boys/girls)	5.1	11.9	7.8	17.6	2.9	7.1	<0.001	<0.001
Not abdominal obesity	WHtR<0.5	4.8	9.5	7.3	14.1	2.7	5.6	<0.001	<0.001
Abdominal obesity	WHtR≥0.5	0.3	2.4	0.6	3.4	0.2	1.4	NS	<0.050
**Obesity^1,2^**									
Normal-fat	FMI<4.58/7.76 kg/m^2^ (boys/girls)	2.3	0.3	1.3	0.4	3.0	0.2	NS	NS
Overfat	FMI≥4.58/7.76 kg/m^2^ (boys/girls)	13.2	5.8	17.0	6.1	10.0	5.6	<0.001	NS
Not abdominal obesity	WHtR<0.5	6.5	1.2	9.5	1.5	4.0	1.0	<0.001	NS
Abdominal obesity	WHtR≥0.5	6.6	4.6	7.4	4.6	5.9	4.7	NS	NS

Abbreviations: BMI, body mass index; FMI, fat mass index; WHtR, waist-to-height ratio; IOTF, International Obesity Taskforce; WHO, World Health Organization. Adolescents were classified on the basis of their BMI using the ^1^WHO-2007 and ^2^IOTF international references. ^1^WHO-2007 [4] cut-offs: normal-weight, BMI-for age and sex <P85; overweight, BMI-for age and sex ≥P85–<P95; obesity, BMI-for age and sex ≥P95. ^2^IOTF [3] cut-offs; normal-weight, BMI for age and sex <25 kg/m^2^; overweight, BMI-for age and sex ≥25-BMI-<30 kg/m^2^; obesity, BMI for age and sex ≥30 kg/m^2^. Significant differences (boys vs. girls) by χ^2^ using ^3^WHO-2007 and ^4^IOTF references. NS: not significant.

Therefore, results showed that among normal-fat adolescents, 91.3% were normal-weight and 8.4% were overweight using the IOTF cut-offs; whereas among overfat adolescents 29.3% were obese, 60.1% overweight and 10.6% normal-weight. Using the WHO-2007 cut-offs among normal-fat adolescents, 88.3% were normal-weight, 8.9% overweight and 2.9% obese; while among the overfat adolescents, 66.7% were obese, 25.8% overweight and 7.6% normal-weight. Among adolescents with abdominal obesity, 20.2% and 79.3% were overweight and obese using the IOTF cut-offs, respectively; whereas 5.2% were overweight and 92.2% obese using the WHO-2007 cut-offs. Independently of the BMI cut-offs used, 2.6% of adolescents with abdominal obesity were normal-weight (data not shown).

On the basis of our results we proposed a new classification for adolescents based not only in body weight (BMI), but also adiposity (FMI) and fat distribution (WHtR): the Adiposity & Fat Distribution classification (AFAD-A) for adolescents (Table 3). In this classification, obesity was defined as a BMI-for age and sex ≥30 kg/m^2^ [3]; whereas normal-weight and overweight groups obtained by IOTF cut-offs were subgrouped according to presence or absence of overfat by FMI using the sex-specific cut-offs proposed by Alvero-Cruz et al. [18] for adolescents into four groups as follows: normal-weight normal-fat, normal-weight overfat, overweight normal-fat and overweight overfat. The overweight overfat and obesity groups were subgrouped according to presence or absence of abdominal obesity using a WHtR cut-off of 0.5 [19]. Less than 1% of normal-weight normal-fat and overfat adolescents had abdominal obesity and were not included in the AFAD-A classification.

**Table 3:** A proposal classification of adolescents according to their body weight (BMI), adiposity (FMI) and fat distribution (WHtR): the Adiposity & Fat Distribution classification for adolescents (AFAD-A classification)^1^.

Body composition markers	
Categories	Cut-offs
**Normal-weight**	BMI-for age and sex<25 kg/m^2^
Normal-fat	Boys: FMI<4.58 kg/m^2^
	Girls: FMI<7.76 kg/m^2^
Overfat	Boys: FMI≥4.58 kg/m^2^
	Girls: FMI≥7.76 kg/m^2^
**Overweight**	BMI-for age and sex ≥25–<30 kg/m^2^
Normal-fat	Boys: FMI<4.58 kg/m^2^
	Girls: FMI<7.76 kg/m^2^
Overfat	Boys: FMI≥4.58 kg/m^2^
	Girls: FMI≥7.76 kg/m^2^
Type-I	WHtR<0.5
Type-II	WHtR≥0.5
**Obesity**	BMI-for age and sex ≥30 kg/m^2^
Type-I	WHtR<0.5
Type-II	WHtR≥0.5

Abbreviations: BMI, body mass index; FMI, fat mass index; WHtR, waist-to-height ratio. ^1^The AFAD-A classification was developed using the International Obesity Task Force (IOTF) cut-offs for BMI categories [3].

**Figure 1:**
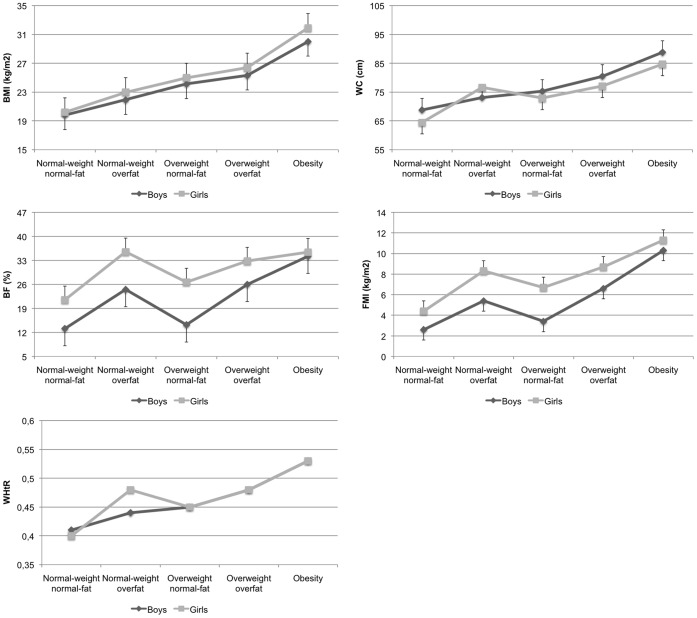
BMI, WC, WHtR, %BF and FMI (mean values ± SD) by the AFAD-A classification. Analysis was adjusted by age.

Figure 1 summarizes anthropometric measurements for the five main groups of the AFAD-A classification. Overall, results showed that whereas normal-weight normal-fat adolescents had lower means for BMI, WC, WHtR, %BF and FMI than their counterparts, normal-weight overfat boys showed lower mean BMI but higher means for %BF and FMI than their overweight normal-fat counterparts. The overweight overfat group also showed higher means BMI, WC, WHtR, %BF and FMI than the overweight normal-fat group but lower means than the obesity group.

## Discussion

Overall, excessive weight (overweight plus obesity) was 27.7% using the WHO-2007 standard and 24.7% using the IOTF reference; whereas the prevalence of overfat by FMI using the cut-offs proposed by Alvero-Cruz et al. [18] was 19.8%; and 7.7% of adolescents had abdominal obesity. Using the IOTF cut-offs, while almost all obese adolescents were overfat (95.1%) and most of them had abdominal obesity (78.7%); about 36% of overweight adolescents were misclassified on the basis of the BMI alone –being higher among girls (56.7%) than boys (17%)-. Using the WHO cut-offs, 85.2% of obese adolescents were overfat and half of them had abdominal obesity; whereas 58.2% of overweight adolescents were normal-fat –being also higher among girls (73.4%) than boys (43.9%)-. Among the normal-weight group, using both IOTF and WHO-2007 cut-offs about 1.4–2.8% of adolescents were overfat, which was higher among boys (4–6%) than girls (<1%).

The present results agree with previous studies [2], [5]–[7] which have pointed out that the IOTF reference and the WHO standard yield different results in terms of prevalence of overweight and obesity. A previous study conducted in 2004 among Canadian children and youth (n = 8661, 2- to 17-year-olds) [5] which compared prevalence estimates of excess weight according to three sets of BMI reference cut-offs: WHO, IOTF and the US Centers for Disease Control (CDC), found that prevalence estimate for the combined overweight/obese category was higher (35%) when based on the WHO cut-offs compared with the IOTF (26%) or CDC (28%) cut-offs. Estimates of the prevalence of obesity were similar based on WHO and CDC cut-offs (13%), but lower when based on IOTF cut-offs (8%). In the same line, a study conducted among Portuguese children and adolescents aged 10- to 18-year-olds (n = 22048), the prevalence of overweight and obesity based in the IOTF cut-offs was 17.0 and 4.6% in girls, and 17.7 and 5.8% in boys, respectively; whereas WHO cut-offs resulted in overweight and obesity prevalence scores of 23.1 and 9.6% in girls, and 20.4 and 10.3% in boys, respectively [6]. Monasta et al. [2] have indicated that these different results are due to the different approaches used to define cut-offs and to the different criteria used to select the samples. Monasta et al. [2] also suggested that at the moment, IOTF reference and cut-offs could be preferable to identify overweight and obesity both at individual and population level because they are at least based on a crude association with ill health later in life, namely the definition of overweight and obesity at 18 years. However, it is important to note that in the present study both international references also provide high different results than the FMI using the cut-offs proposed by Alvero-Cruz et al. [18]. Of course BMI and FMI are different terms and although BMI performs moderately well as a proxy for these indicators, particularly at the upper end of the distribution curve [11], an important percentage of subjects classified as overweight or obese did not have really excess adiposity [20], percentage which may depend on the reference used.

The ideal definition of overweight and obesity would be based either on a close correlation with indicators of future cardiovascular and metabolic disease or on their ability to predict adverse future health outcomes [11]. Therefore, despite that BMI have been suggested as a good proxy for the screening of excess body fat in adolescents when considering a whole population, as in clinical settings others criteria may be also useful in epidemiological studies in which anthropometric measurements are used to screen overweight and obesity prevalence. Moreover, a single definition useful in both epidemiological and clinical settings should be achieved because epidemiological studies should determine the magnitude of the overweight and obesity problem in a population and also to stress the need for lifestyle changes while not exaggerating risks of future obesity and cardiovascular disease.

Because it is difficult to exclude BMI from the normal-weight and obesity definition despite that provides no information regarding the composition of the weight, or its distribution; FMI and WHtR could combine BMI for a better screening and surveillance. The FMI is a useful measure to evaluate body composition parameters by effectively eliminating differences in body fat associated with height [12]. Alvero-Cruz et al. [18] derived cut-off points for FMI from a sample of Spanish adolescents (150 subjects, 75 boys and 75 girls) showing that the FMI had higher accuracy for overweight screening than BMI. Their results pointed out in boys that predictive positive value (meaning the diagnosis of excessively fat adolescent as overweight) were 78.1% for BMI and 89.2% for FMI; and predictive negatives value (meaning the diagnosis of lean adolescent as non-overweight) were 81.4% for BMI and 100% for FMI in them. In girls, predictive positive value for BMI were 34.8%, predictive positive value for FMI were 81.4%; and predictive negative value for BMI and FMI were 98.1% and 100%, respectively. Combining BMI and FMI, our results suggest that IOTF cut-offs have high specificity for obesity –more than WHO cut-offs-, in our adolescent population. However, there was much less evidence on the optimal definition of being normal-weight or overweight. Therefore, the present results supported that a cut-off BMI≥30 kg/m^2^ for age and sex [3] may be a good proxy for obesity in boys and girls, and also a cut-off BMI<25 kg/m^2^ [3] for age and sex for normal-weight in girls; whereas FMI may reduce misclassification among normal-weight boys and overweight adolescents. Thus, adolescents may be classified into five main groups as follows: normal-weight normal-fat, normal-weight overfat, overweight normal-fat, overweight overfat and obese.

It is also well established that central or visceral obesity is a major factor for the clustering of cardiovascular risk factors which defines the metabolic syndrome [21]. The determination of adolescents with abdominal obesity in both overweight and obesity status could useful to identify adolescents who being overweight or obese have higher probability for cardiovascular risk factors. Thus, overweight overfat and obesity adolescents could be classified into type-I and type-II according to the absence or presence of abdominal obesity, respectively. Abdominal obesity should be assessed by WHtR which has been proposed because of its ability to predict cardiovascular risk factors [22]–[25] and to estimate abdominal fat distribution [26], particularly in individuals who may not be classified as overweight or obese by BMI [16], [18], [19], [27].

It is important to note that direct measurement of adiposity with sophisticated techniques is considered to be superior to indirect measures [28]. However, in many circumstances it is more desirable to utilize widely available and simple techniques such as anthropometry. Therefore, it should be recommended that not only BMI but also FMI and WHtR be used whenever possible in both clinical and epidemiological settings. Both, anthropometric measurements are quick, cheap and simple which require only limited training and standardized assessment to obtain reliable data [17]. Moreover, FMI and WHtR are normalized for body size making comparisons between individuals or populations, or within individuals or populations over time, to be meaningful [29].

The contribution of this research may lead to better methods for measuring normal-weight and overweight to support this area of public health research, at least for a more accurate classification of Spanish adolescents because further research will be needed to evaluate the FMI cut-offs proposed by Alvero-Cruz et al. [18] to be generalized for international use. On the basis of our results, adolescents may be classified not only by body weight (BMI) but also adiposity (FMI) and fat distribution (WHtR). We proposed a new classification for adolescents, the Adiposity & Fat Distribution classification (AFAD-A) which classifies adolescents into the following groups: (1) normal-weight normal-fat; (2) normal-weight overfat; (3) overweight normal-fat; (4) overweight overfat (type-I and type-II, depending on the presence or absence of abdominal obesity, respectively), and (5) obesity (type-I and type-II). To facilitate the work of clinicians and epidemiologists, a questionnaire summarized as the AFAD-A classification is proposed (Table 4), despite that further research will be needed to evaluate its utility.

**Table 4:** A proposal questionnaire to classify adolescents according to their body weight, adiposity and fat distribution: the AFAD-A questionnaire classification.

Question	Result
1. What body mass index (BMI, kg/m^2^) has the subject according to their sex and age (applied Cole et al,2000 cut-offs)?	
a. BMI for age and sex <25 kg/m^2^ (go to *question 2*)	***Normal-weight***
b. BMI for age and sex ≥25 e IMC<30 kg/m^2^ (go to *question 3*).	***Overweight***
c. BMI for age and sex ≥30 kg/m^2^ (go to *question 4*).	***Obesity***
2. If they are **normal-weight**: What fat mass index (FMI, kg/m^2^) has the subject according to their sex?	
a. Boys: FMI<4.58 kg/m^2^; girls: FMI<7.76 kg/m^2^.	***Normal-weight normal-fat***
b. Boys: FMI≥4.58 kg/m^2^; girls FMI≥7.76 kg/m^2^.	***Normal-weight overfat***
3. If they are **overweight**: What fat mass index (FMI, kg/m^2^) has the subject according to their sex?	
a. Boys: FMI<4.58 kg/m^2^; girls: FMI<7.76 kg/m^2^.	***Overweight normal-fat***
b. Boys: FMI≥4.58 kg/m^2^; girls FMI≥7.76 kg/m^2^ (go to *question* 4).	***Overweight overfat***
4. If they are **overweight at risk** or **obesity**: What waist-to-height ratio (WHtR) has the subject(independently of their sex and age)?	
a. WHtR<0.5.	***Overweight overfat*** * or * ***Obesity type-I***
b. WHtR≥0.5.	***Overweight overfat*** * or * ***Obesity type-II***

### Conclusions

There are differences between IOTF and WHO-2007 international references and there is a misclassification when adiposity is considered. Surveillance, prevention and treatment of childhood and adolescent obesity require methods of defining obesity that are simple enough to be practical in most clinical and public health settings, but are also valid [30], [31]. However, identification of adolescents in normal-weight and overweight with excess body fat is also important not only because they have some increased risk of adiposity-related comorbid conditions [8], but also psychosocial complications derived from body fatness [32]. Therefore, achieving a reliable and accurate estimation of body fatness and fat distribution is essential in both clinical and epidemiological settings. Our results support that it should be recommended that not only BMI but also FMI and WHtR be used whenever possible in both clinical and epidemiological settings.

Despite that further research will be needed to evaluate the utility of the AFAD-A questionnaire and classification, it could be the starting point towards an improvement in the traditional definition of normal-weight, overweight and obesity which may be a useful tool to surveillance adolescent’s overweight on clinical and epidemiologic settings.

### Limitations of the Study

The cut-offs point considered for FMI were proposed by Alvero-Cruz et al. [18] and derived from 150 subjects (75 boys and 75 girls). However, as it has been indicated by Alvero-Cruz et al. [18] the average values of basic anthropometric variables (weight, height and BMI) of other 450 subjects were not significant different from those of the sample assessed, indicating that these cut-offs should be useful for overweight diagnostic in Spanish adolescents.

Certainly, it is important to note that to calculate the FMI from anthropometric measures there is an intermediate step consisting of applying an equation that allows determining the percentage of body fat, and the value depends on the applied equation that increase error misclassification. Rodríguez et al. [17] compared the most commonly used equations to predict body fatness from skinfold thickness with dual-energy X-ray absorptiometry (DEXA) and found that most equations did not demonstrated good agreement compared with DEXA. However, they proposed that Slaughter et al. [16] equations may be used in adolescents from both sexes to predict BF when a relative index of fatness is required in field or clinical studies. Nevertheless, the present study did not take into account pubertal development despite that chronological age may vary dramatically during this phase. Adolescents have been classified according to their pubertal stage, boys were divided into two groups: pubertal (12 to 14 y-o) and post-pubertal (15 to 17 y-o) [33].

Despite there is widely accepted that WHO and IOTF definitions have several limitations to define overweight and obesity and also yield different results in terms of prevalence of overweight and obesity on the same dataset, there are insufficient data to substitute these definitions for other anthropometric measurements. However, the combination of BMI and subcutaneous fat is intended to maximize specificity in identifying those adolescents who are normal-weight normal-fat or overfat, and overweight normal-fat or overfat. None the less, recommendation of AFAD-A for adolescent body composition classification must be considered as provisional because of inadequate evidence of the %BF and FMI derived from anthropometric measurements are better measurements than that from WHO or IOTF definition. Other techniques for body composition assessment such as densitometry, dual-energy X-ray absorptiometry, and magnetic resonance imaging provide more accurate information on fat and lean masses; however, they are expensive and impractical for use in routine clinics and epidemiological studies [34]. Bioelectrical impedance analysis (BIA), on the other hand, is relatively cheap and easy to use [34]. Direct measures should be used as a gold standard to validate indirect (anthropometric) measures of body fatness. Full studies are required before the recommendations for AFAD-A classification can be considered more than provisional.
